# Supplementary material for: Influence of Different Pt Functionalization Modes on the Properties of CuO Gas-Sensing Materials
Source: Sensors (Basel). 2023 Dec 25;24(1):120. doi: 10.3390/s24010120 (PMC10780899; doi:10.3390/s24010120)
Supplement: Supplementary file 1 [file sensors-24-00120-s001.zip › Supporting Information.pdf]

Supporting information for

Influence of different Pt functionalization modes on the properties of CuO gas sensing materials

Xiangxiang Chen [a,b,c], Tianhao Liu [a], Yunfei Ouyang [a], Shiyi Huang [a], Zhaoyang Zhang [a], Fangzheng Liu [a], Lu Qiu [a], Chicheng Wang [a], Xincheng Lin [a], Junyan Chen [a], Yanbai Shen [c*]

[a] Zijin School of Geology and Mining, Fuzhou University, Fuzhou 350108, China

[b] Fujian Key Laboratory of Green Extraction and High Value Utilization of New Energy Metals, Fuzhou 350108, China

[c] School of Resources and Civil Engineering, Northeastern University, Shenyang 110819, China

*Corresponding author.
E-mail address: shenyanbai@mail.neu.edu.cn (Y. Shen).

Table of Contents

Figure S1. Response and recovery curves of the sensors based on pristine and Pt-loaded CuO nanorods upon exposure to 5 ppm NO_2 at various operating temperature. (a) Pristine; (b) 1 mol% Pt@CuO; (c) 2 mol% Pt@CuO; (d) 5 mol% Pt@CuO..**S-2**

Figure S2. Response and recovery curves of the sensors based on pristine and Pt-doped CuO nanorods upon exposure to 5 ppm NO_2 at various operating temperature. (a) Pristine; (b) 1 mol% Pt-CuO; (c) 2 mol% Pt-CuO; (d) 5 mol% Pt-CuO..**S-3**

Figure S3. FTIR spectra of (A) Pt-loaded and (B) Pt-doped CuO nanorods with different Pt concentration. (a) 0 mol% Pt; (b) 1 mol% Pt; (c) 2 mol% Pt; (d) 5 mol% Pt.................................**S-4**

Figure S4. Response and recovery curves of the sensors based on 2 mol% (A) Pt-loaded CuO and (B) Pt-doped CuO nanorods upon exposure to 2 ppm NO_2 at 25 °C and different humidity..........**S-5**

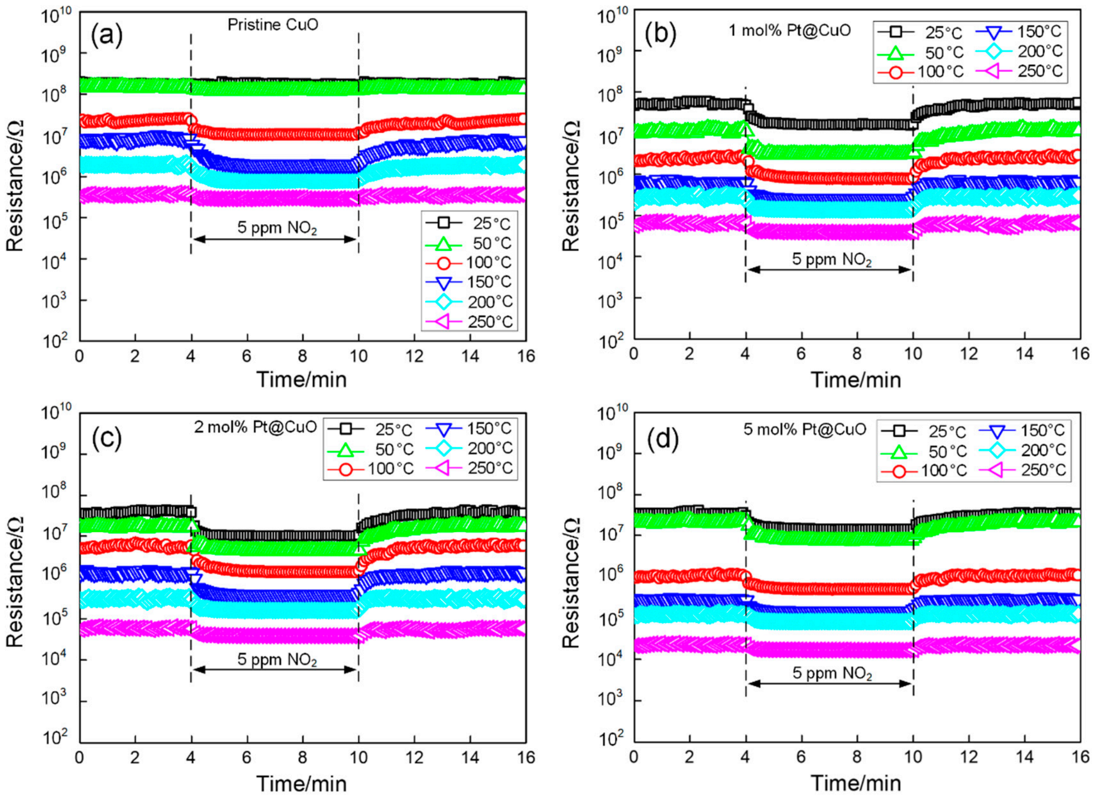

Figure S1. Response and recovery curves of the sensors based on pristine and Pt-loaded CuO nanorods upon exposure to 5 ppm NO_2 at various operating temperature

(a) Pristine; (b) 1 mol% Pt@CuO; (c) 2 mol% Pt@CuO; (d) 5 mol% Pt@CuO

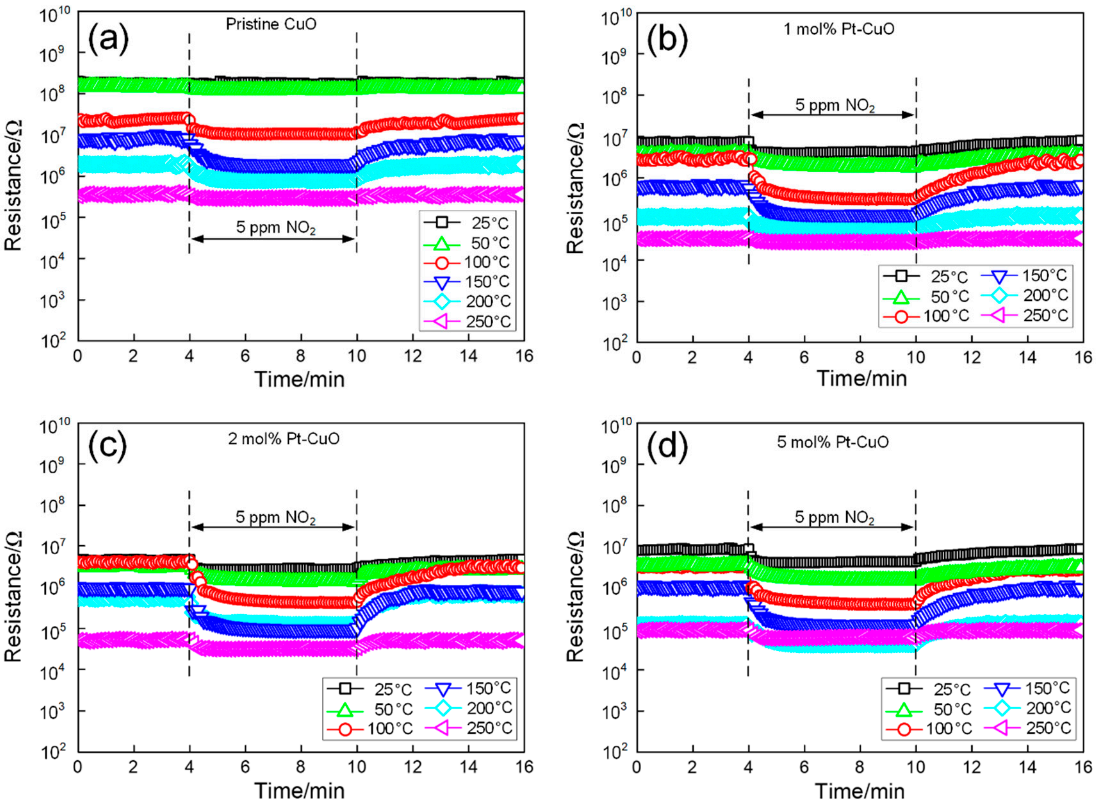

Figure S2. Response and recovery curves of the sensors based on pristine and Pt-doped CuO nanorods upon exposure to 5 ppm NO_2 at various operating temperature

(a) Pristine; (b) 1 mol% Pt-CuO; (c) 2 mol% Pt-CuO; (d) 5 mol% Pt-CuO

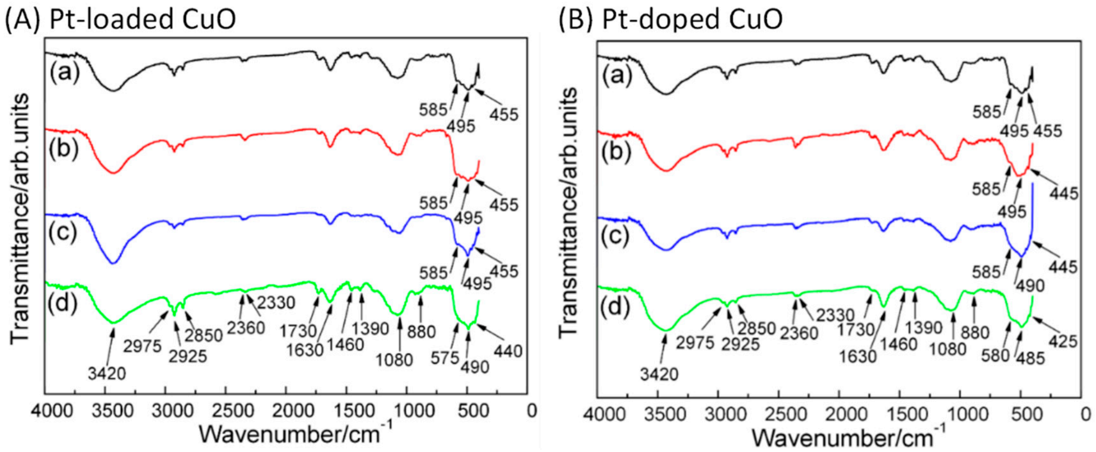

Figure S3. FTIR spectra of (A) Pt-loaded and (B) Pt-doped CuO nanorods with different Pt concentration

(a) 0 mol% Pt; (b) 1 mol% Pt; (c) 2 mol% Pt; (d) 5 mol% Pt

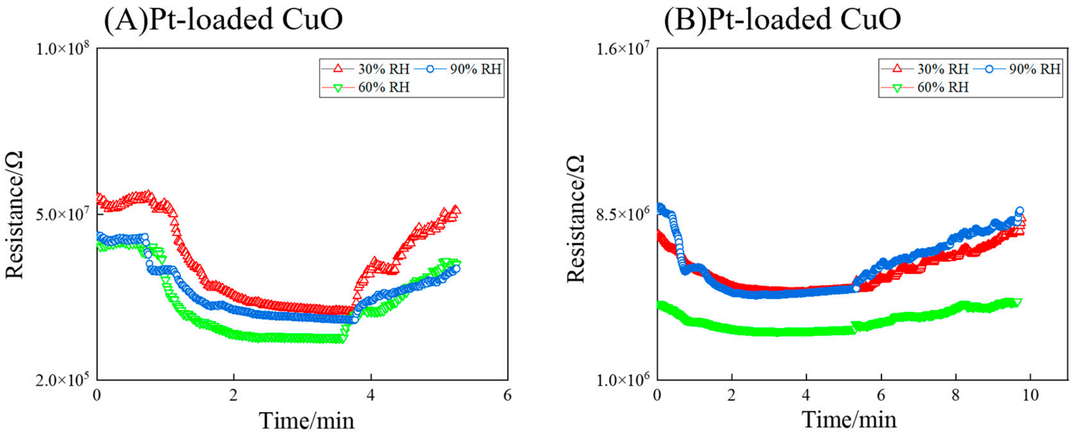

Figure S4. Response and recovery curves of the sensors based on 2 mol% (A) Pt-loaded CuO and (B) Pt-doped CuO nanorods upon exposure to 2 ppm NO_2 at 25 ℃ and different humidity
